# Nanotechnology in the Diagnosis and Treatment of Osteomyelitis

**DOI:** 10.3390/pharmaceutics14081563

**Published:** 2022-07-27

**Authors:** Demi Zapata, Jordan Higgs, Hunter Wittholt, Kishore Chittimalli, Amanda E. Brooks, Pranothi Mulinti

**Affiliations:** 1 College of Osteopathic Medicine, Rocky Vista University, Ivins, UT 84738, USA; demi.zapata@rvu.edu (D.Z.); jordan.higgs@rvu.edu (J.H.); hunter.wittholt@co.rvu.edu (H.W.); abrooks@rvu.edu (A.E.B.); 2 Department of Pharmaceutical Sciences, North Dakota State University, Fargo, ND 58108, USA; kishore.chittimalli@ndsu.edu

**Keywords:** osteomyelitis, bone infection, nanotechnology, nanoparticles, nanobiopolymers

## Abstract

Infection remains one of the largest threats to global health. Among those infections that are especially troublesome, osteomyelitis, or inflammation of the bone, typically due to infection, is a particularly difficult condition to diagnose and treat. This difficulty stems not only from the biological complexities of opportunistic infections designed to avoid the onslaught of both the host immune system as well as exogenous antibiotics, but also from changes in the host vasculature and the heterogeneity of infectious presentations. While several groups have attempted to classify and stage osteomyelitis, controversy remains, often delaying diagnosis and treatment. Despite a host of preclinical treatment advances being incubated in academic and company research and development labs worldwide, clinical treatment strategies remain relatively stagnant, including surgical debridement and lengthy courses of intravenous antibiotics, both of which may compromise the overall health of the bone and the patient. This manuscript reviews the current methods for diagnosing and treating osteomyelitis and then contemplates the role that nanotechnology might play in the advancement of osteomyelitis treatment.

## 1. Introduction

Bone is naturally a highly vascularized connective tissue with a distinct angiogenic pattern that often develops simultaneously to bone mineralization [1]. However, upon aging, trauma, or infection, the vasculature of bone can be compromised, leading to an almost idealized niche for opportunistic bacterial adhesion and growth. Not only does this compromised vasculature provide a favorable microenvironment (i.e., lower oxygen, favorable pH, etc.) for bacterial growth, but it also inhibits the delivery of antibiotic. This combination of conditions leads to bone infections such as septic arthritis, spinal infections, osteomyelitis, and diabetic foot osteomyelitis, that can cause severe trauma and may lead to permanent disabilities. The clinical management of such infections may require a new approach for both rapid diagnosis and treatment.

## 2. Osteomyelitis

**Figure 1:**
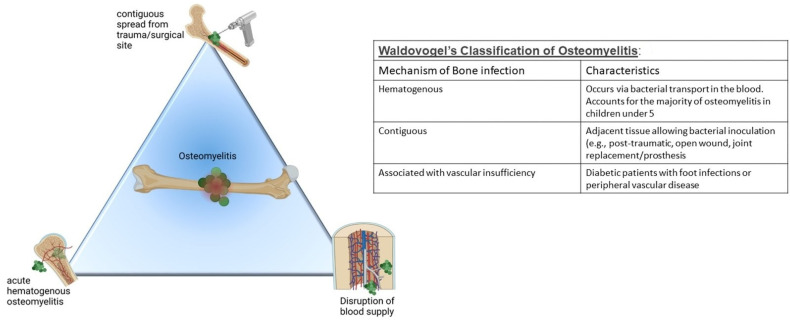
Osteomyelitis arises in one of three primary ways. Regardless of the underlying cause, vascular disruption due to the formation of sequestra can occur, making treatment very difficult (Lima et al.). Waldvogel’s classification of osteomyelitis [3] describes the characteristics of each of these ways. Figure created with BioRender.com.

Osteomyelitis is defined as inflammation of the bone, usually due to infection of the bone marrow and adjacent osseous structures with the possibility of affecting encompassing surrounding soft tissue [2]. *Staphylococci* collectively cause up to 75% of these infections with *S. aureus* being the main pathogen in 30% to 60% of cases [3]. Importantly, non-infection osteomyelitis can also occur in an autoinflammatory disease, chronic noninfectious osteomyelitis (CNO). While the clinical presentation may appear similar to infectious osteomyelitis, CNO seems linked to a genetic cause (recently reviewed by Hedrich et al. [4]) and occurs rarely [5]; hence, the current review focuses on the more common infectious osteomyelitis. Infectious osteomyelitis has a high recurrence rate and chronicity due to the ability of *Staphylococcus aureus* to infiltrate osteoblasts through cell surface fibronectin-binding protein and α5β1 integrin [6], thereby avoiding both antibiotic and host immune onslaughts [7], leading to devastating effects. Osteomyelitis can be divided into various types (1) acute hematogenous osteomyelitis caused by the infection spreading through the bloodstream (more commonly seen in children under 17 years); (2) secondary osteomyelitis resulting from contiguous spread sourced from adjacent infection sites (such as those from trauma or surgery) or an orthopedic implant [8]; or (3) secondary osteomyelitis due to vascular insufficiency or neuropathy as seen in diabetic foot ulcers (Figure 1) [9,10]. 

## 3. Pathophysiology of Osteomyelitis

**Figure 2:**
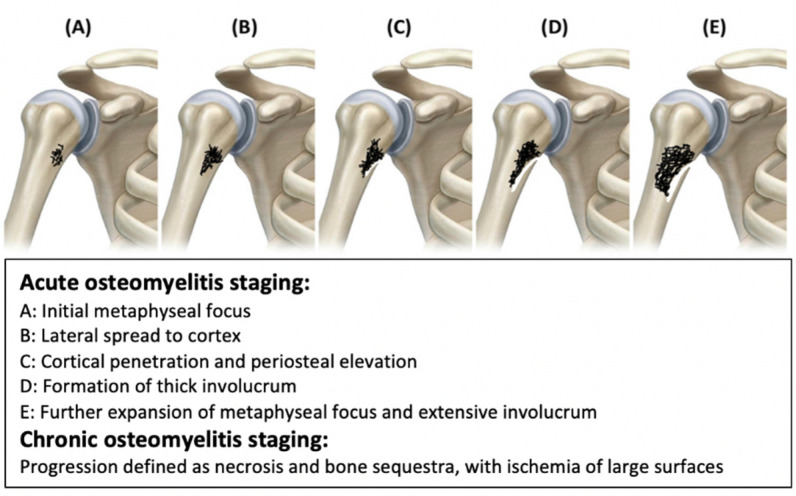
Progression of either (**A**) acute or (**B**) chronic osteomyelitis (**C**) Cortical penetration and periosteal elevation (**D**) Formation of thick involucrum (**E**) Further expansion of methaphyseal focus and extensive involucrum. Two significant differences between acute and chronic osteomyelitis should be noted: the formation of a biofilm and the infiltration of the bacteria into host cells, both of which happen in chronic osteomyelitis. Modified from Desimpel et al. [2].

The pathophysiology and etiology of osteomyelitis is dependent on the underlying source of the infection, the location of the bone, and the structural difference of the bone based on the age of the patient. Recent studies have suggested that not only does the mineral structure of bone change based on age, but the vascular structure is also altered; a change that seems to be driven by impaired endothelial Notch signaling [11,12]. This alteration in blood flow may compromise osteogenesis and the ability of bone to heal, potentially predisposing it to infection, particularly after trauma, surgery, or injury [13]. Based on the critical role that vasculature plays in bone regeneration, it is clear how both contiguous spread and disruption of the blood supply lead to osteomyelitis, but osteomyelitis due to acute hematogenous spread of infection in pediatric patients does not initially seem to fit that pattern. However, upon closer consideration, acute hematogenous osteomyelitis is often associated with the venous architecture in pediatric patients, which is associated with more turbulent blood flow that occurs at the metaphyseal vessel loop, such as that found near the lumbar spine and growth pates of pediatric patients [9]. Combined with a transient bacteremia often resulting from trauma, this turbulent flow causes a venous pooling of blood near the metaphysis of bone, allowing deposition of bacteria near the growth plate and initiating an osteomyelitic progression (Figure 2) [2]. Regardless of the pathophysiology of osteomyelitis, diagnosis of the disease can be particularly difficult.

## 4. Diagnosis

Accurate and timely diagnosis of osteomyelitis has proven difficult in the medical community. The diagnosis of osteomyelitis is complicated by its heterogeneous nature, often non-specific clinical presentation, and the limited sensitivity of any single diagnostic procedure. Clinical presentation of osteomyelitis may vary based on location and could include “acute, abrupt local symptoms of systemic toxicity; or insidious onset of vague pain over the site of infection, progressing to local tenderness and constitutional symptoms (fever, malaise, anorexia, night sweats)” [14]. Although diagnosis of osteomyelitis is less than definitive, with staging and classification of osteomyelitis facing challenges and discrepancies based on the anatomical location of disease and clinician experience, it is clear, as with most disease processes, that early diagnosis and intervention is key. Several different classifications have been proposed to provide clinical guidance during diagnosis and staging. Schmidt et al. proposed a diagnostic scoring system in 2011, that combines the results of five different diagnostic procedures to give an overall score and confidence classification for the diagnosis of osteomyelitis (Table 1) [15]. However, as Tiemann et al. pointed out, such a scale is still not definitive due to inherent weaknesses in the specificity and sensitivity of the available diagnostic techniques [16]. The utility of each of these diagnostic procedures is nicely summarized by Tiemann et al. [16] and is briefly outlined below:

- *Clinical history and risk factors:* Clinical history and risk factors for acute hematogenous osteomyelitis (AHO) include trauma, sepsis, bacteremia, chronic catheterization, immunodeficiency [17]. These same factors should be considered for other forms of osteomyelitis as well as including assessment of vascular disease. This criterion cannot be solely relied on for diagnosis as it requires self-reporting, which may compromise the validity.
- *Clinical examination:* Clinical exams often reveal diffuse or non-specific findings, making diagnosis on this basis alone virtually impossible. Nevertheless, AHO should be considered if a child presents with a fever and localized bone pain [17].
- *Laboratory test results*: The presence of elevated inflammatory markers are too non-specific to explain the differential diagnoses as osteomyelitis [18]; however, although it is not definitive for diagnosis, it is atypical for patients with acute osteomyelitis to have a normal erythrocyte sedimentation rate (ESR) or normal levels of C-reactive protein (CRP) [9].
- *Imaging*: X-ray, although not the gold standard, is one of the first diagnostic tools used to evaluate osteomyelitis. Unfortunately, radiographs may be unremarkable for 10–14 days following bone infection, with adults experiencing a longer delay [19]. Consequently, by the time that conventional radiographs effectively demonstrate lytic changes, 50–75% of the bone matrix has been destroyed [20]. This makes early detection of osteomyelitis unlikely. Nevertheless, X-ray radiographs are often initially performed to rule out alternative and more common diagnoses such as fracture or malignancy. Notably, several retrospective studies have revealed that X-ray imaging, once thought to be the gold standard, is of moderate diagnostic accuracy when detecting factures [21]. Alternatively, fluorodeoxyglucose-positron-emission-tomography/computed tomography (_18_F-FDG-PET/CT) may prove more specific, particularly when used as a hybrid technique with a radioisotope. _18_F-FDG-PET/CT has proven useful specifically in patients with MRI contraindications and in the diagnosis of vertebral osteomyelitis as well as in the detection of other metastatic sites of infection [18]. However, PET scans also have limited specificity due to radionuclide uptake that can be present in many inflammatory and neoplastic processes [22]. Magnetic resonance imaging (MRI) has a specificity of 90% and may allow earlier diagnosis of infection due to its ability to image soft tissue prior to bone infiltration.
- *Microbiology:* Microbiological tests often result in false-negative outcomes due to the location of causative pathogens, the possibility of the patient starting antibiotic therapy prior to bone biopsy, and the culturability of the organism [23,24,25]. Thus, the recommendation is that isolation of causative microorganisms should be attempted using a minimum of three bone tissue cultures and the use of modern molecular testing should be employed [26].
- *Histopathology*: Bone biopsy (bone scintigraphy) at the site of necrosis is the gold standard of osteomyelitis diagnosis. Positive osteomyelitis bone biopsy results typically include elevated C-reactive protein (CRP), erythrocyte sedimentation rate (ESR), with presence of causative organism (typically *S. aureus*). Bone scintigraphy in conjunction with a gallium scan can assist in the localization of the infected bone.

## 5. Vertebral Osteomyelitis—A Case Study to Demonstrate the Diagnostic Difficulties Associated with Osteomyelitis

The widespread nature of back pain and the variety of underlying causes poses a significant challenge when narrowing down the differential diagnoses to vertebral osteomyelitis. However, vertebral osteomyelitis (AKA spinal osteomyelitis or spondylodiscitis), which typically manifests as back pain with absence of other associated symptoms, accounts for 3–5% of osteomyelitis cases [22]. Risk factors for vertebral osteomyelitis include advanced age, diabetes, IV-drug or long-term corticosteroid use, endocarditis, and immunocompromised individuals [22,27]. A 2020 study by Fragío Gil et al. analyzed 116 patient records to assess clinical diagnosis and treatment regimens. The average patient was middle aged with a history of subacute back pain and inconsistently presented with a fever and/or neurological damage at diagnosis [27]. The typical etiopathogenesis of the vertebral osteomyelitis begins with either spinal trauma or postoperative hematogenous spread. Following the introduction of the infectious pathogen into the vertebral column, it spreads to the surrounding paraspinal tissues, nerve roots, epidural space, and intradural space [22]. Infection associated with vertebral osteomyelitis often transverses the disc space, which encompasses the surrounding vertebral body [14]. Osseous and soft tissue destruction may occur due to the ensuing inflammation and abscess formation. Abscesses of the anterior paraspinal space or anterior epidural space with associated soft tissue edema may cause spinal compression (nerve root and/or spinal cord) which if not timely diagnosed and treated, may be accompanied with motor weakness and in severe cases paralysis and are a common clinical features of vertebral osteomyelitis [22].

Diagnosis of vertebral osteomyelitis traditionally is initiated due to back pain and typically begins with imaging (e.g., X-ray) to rule out more common diagnoses, although it should be noted that X-ray is often an unreliable modality for diagnosing vertebral osteomyelitis diagnosis due to lack of sensitivity for the pathology [22]. MRI is useful in patients with vertebral osteomyelitis, particularly if an epidural abscess is suspected. The MRI results of a patient with vertebral osteomyelitis commonly show intervertebral disc infection, in which the infection has spread to the two adjacent vertebral body endplates, which may collapse in a chronic infection. However, if imaging suggests vertebral osteomyelitis but blood cultures are negative, the causative microorganism must be isolated prior to initiating antibiotic therapy. CT-guided percutaneous biopsy is the modality of choice to aspirate a sample of the affected bone to identify the causative microorganism. Febrile patients who present with back pain often receive a complete blood cell count (CBC) test, in addition to both aerobic and anaerobic blood cultures [22]. Unfortunately, as with other osteomyelitis locations, CBC’s possess low sensitivity for vertebral osteomyelitis as is demonstrated by neutrophil counts, which would be expected to be relatively low; however, around 40% of those affected by vertebral osteomyelitis have white blood cell counts within normal limits [22]. As with other forms of osteomyelitis, patients with vertebral osteomyelitis also present with elevated inflammatory marks of C-reactive protein (CRP) and erythrocyte sedimentation rate (ESR).

## 6. Osteomyelitis Classification and Staging

**Figure 3:**
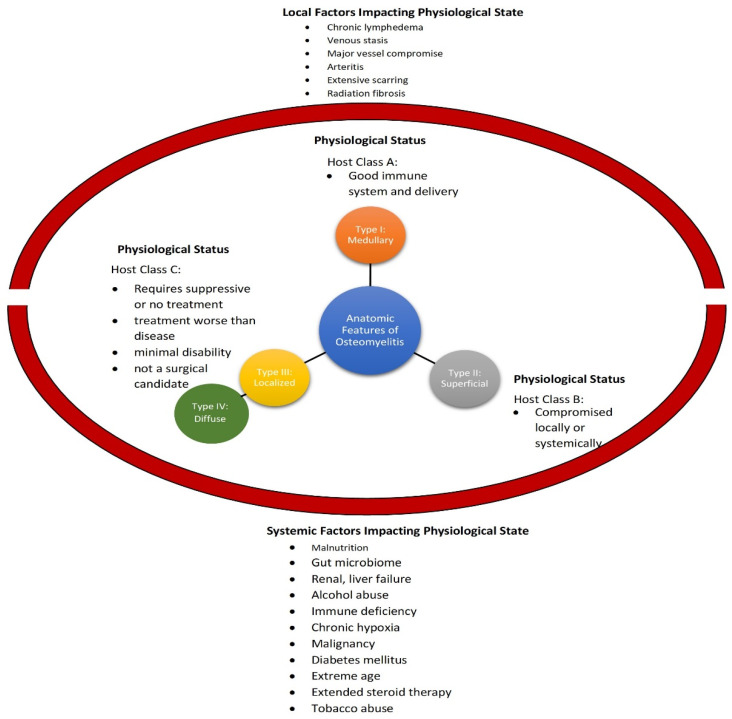
The Cierny and Mader classification reproduced [29].

Historically, there have been multiple independent efforts to classify and stage osteomyelitis so as to improve the consistency of diagnosis and treatment. A review of this historical progression, although important, is beyond the scope of the current review and the reader is referred to a review from Marais et al. [28]. Currently, there are two primary classifications that are used: the Waldvogel classification of osteomyelitis to determine the underlying mechanism of seeding and the Cierny and Mader classification scale (Figure 3) to assign the osteomyelitis subtype based on a combination of anatomical features and the physiological status of the patient, yielding 12 different combinations. It should be noted that the Cierny and Mader classification is traditionally only used for adult osteomyelitis [29]. The physiological status of the patient is determined using a variety of systemic and local factors. Considering the emerging connection between the gut microbiome and bone regeneration would indicate that gut microbiome dysbiosis should also be considered an important systemic physiological factor. McPherson et al. attempted to make the Cierny and Mader scale more specific and objective by adding measurable criteria [30]. Lautenbach built on this work and developed a staging system integrating clinical, laboratory, and radiological findings to create a graded scale to guide treatment decisions [28]. Importantly, this scale distinguishes between acute and chronic in clinical grades but only chronic in laboratory workups and has no distinction between acute and chronic for radiologic findings (Table 2). Finally, it should be emphasized that the Cierny and Mader scale, the Lautenbach staging method and other similar classifications systems should be employed after diagnosis of osteomyelitis and not in place of diagnostic criteria such as the Schmidt et al. criteria shown in Table 1.

## 7. Acute Osteomyelitis

Acute osteomyelitis is often staged according to the progress of infection invasion throughout the bone tissue. Postoperative patients with acute post-traumatic osteomyelitis typically present with signs of local hyperemia, pain, inflammation, elevated exudate and suspicion of hematoma at the surgical site, which usually manifests within two weeks after exposure to the bacteria [10]. Additionally, there may be a dull pain with or without motion and sometimes constitutional symptoms such as fever or chills. If the infection occurs in the presence of a prosthesis, the surgeon must act quickly to perform routine debridement and obtain blood/bone cultures in order to salvage the implant and potentially the residual limb [10]. Acute osteomyelitis may also present as septic arthritis, especially if the metaphysis of the bone is within the infected joint capsule. Septic arthritis of the elbow, shoulder, and hip joints may complicate osteomyelitis of the proximal radius, humerus, and femur, respectively. New or worsening neck or back pain in a patient with fever, elevated inflammatory markers (CRP, erythrocyte sedimentation rate [ESR]), bacteremia or endocarditis should raise the suspicion for native vertebral osteomyelitis (NVO). The symptomology in subacute presentations is even more vague; some patients may have generalized malaise, mild pain over several weeks with minimal fever, or other constitutional symptoms.

## 8. Chronic Osteomyelitis

In chronic osteomyelitis, symptoms may occur over a longer duration of time, usually more than two weeks. As with acute osteomyelitis, patients may also present with swelling, pain, and erythema at the site of infection, but constitutional symptoms such as fever are less common. Patients who have deep or extensive ulcers that do not heal after several weeks of appropriate therapy, especially in people with diabetes or debilitated patients, should raise the suspicion for osteomyelitis. The complications of progressing diabetes and PVD can hinder patients unaware of open wounds and patients may experience a decline in the body’s ability to self-heal. In fact, the incidence of chronic osteomyelitis is becoming increasingly more common due to the rising prevalence of diabetic foot infections and peripheral vascular disease (PVD) [18] compounded by rising rates of antibiotic-resistant infections and the ability of the primary causative organism, *S. aureus*, to infiltrate host cells. Thus, physical examination should focus primarily on finding a possible nidus of infection, assessing the sensory function, and peripheral vasculature.

## 9. Treatment

Current treatment for osteomyelitis has yet to be optimized; this is potentially a reflection of the heterogeneity of the disease and variability in diagnostic criteria and staging; although it is clear that an effective treatment regimen includes extensive surgical debridement particularly for chronic infections and antibiotic therapy varying from four to six months, depending on the condition. Despite these general guidelines, there is a paucity in the data about proper treatment of osteomyelitis, including the type of antibiotics, treatment duration and the route of administration; most aspects of antibiotic treatment in bone, particularly diseased bone with a compromised vascular supply, are still poorly understood, a problem only compounded by expanding antibiotic resistance. Biofilm formation, which in and of itself can be considered a form of antibiotic resistance, complicates the treatment plan further as the dead bone acts as a surface for the attachment and growth of the bacteria. In chronic osteomyelitis, biofilm development due to *Staphylococcus* is in part to blame for the high persistence and recurrence of infection rate in patients [31]. Thus, the treatment of osteomyelitis is still mostly based on expert opinions. Under current consensus guidelines, parenteral administration of antibiotics remains the mainstay of antimicrobial therapy, with the choice of drug based on bactericidal activity, toxicity and cost. Parenteral therapy is generally recommended for six weeks; however, the length of treatment is arguable as there is no evidence-based rationale for the recommendation. This use of systemic antibiotic therapy has inherent drawbacks, including changes in the systemic concentrations (i.e., fluctuating between non-therapeutic and toxic concentrations) and repeated administration of the antibiotic. One effective and promising way to overcome these drawbacks is local delivery of antibiotics at the site of infection. Local drug delivery also has the advantage of easy penetration into the bone tissue, leading to an increased, stable, localized concentration of antibiotics with reduced toxicity and side effects [32]. Several embodiments of local antibiotic delivery to bone are in clinical practice with several more in the approval pipeline [33].

After proper diagnosis of osteomyelitis, treatment options can take a few modalities. However, direct sampling of any open wound for culture with antimicrobial sensitivity is necessary for accurate treatment protocol. Nevertheless, as previously described, not all microbes are culturable and antimicrobial sensitivity is limited and variable based on the in vitro testing environment. Regardless of their weaknesses, antibiotics are still the first line of treatment. Acute phase osteomyelitis is treated via intravenous antibiotic regimen (Table 3) [14,34]. Alternatively, oral step-down therapy to a rifampin-combination regimen has shown effectiveness for patients who are responding clinically [14]. Novel treatments include daptomycin or linezolid which currently have undefined results [14]. For more complicated or advanced infection with certain clinical presentations, such as spinal cord compression by *staphylococcal* osteomyelitis with associated epidural abscess, abscess formation of paraspinal or psoas origin, or persistent or recurrent infections resistant to medical therapy, surgery may be indicated [22]. Surgery may also be necessary if the infection provokes neurological deficits [22].

While several treatment protocols have been recommended based on the diagnostic criteria and staging, the current treatment for osteomyelitis has yet to be optimized, as many individuals struggle with persistent and/or recurrent infections. *Staphylococcal*-biofilm development in affected bone is in part to blame for the high persistence and recurrence of infection rate in patients with chronic osteomyelitis [35]. *Staphylococcal* biofilms are able to form and thrive in the sequestrum of bone, which has decreased oxygen tension and vascularity [35]. Antibiotic therapy does not efficiently penetrate the biofilm matrix and the internal source of infection. If the sequestrum persists in bone, the infection will continue to spread, leading to the need for extensive debridement procedures and possible limb amputation [35]. In addition to the presence of sequestra, the weakening of bone is commonly linked to increased cytokine production concomitant with infection. Cytokine production facilitates the activity of osteoclasts, which are responsible for bone reabsorption. Compounding the issue, bone formation is also compromised by infection as the result of increasing osteoblast cell death. The treatment of osteomyelitis, particularly extensive surgical debridement, may also lead to bone weakening. These combined factors lead to bone that is prone to damage and subsequent fracture [35].

## 10. Nanotechnology

**Figure 4:**
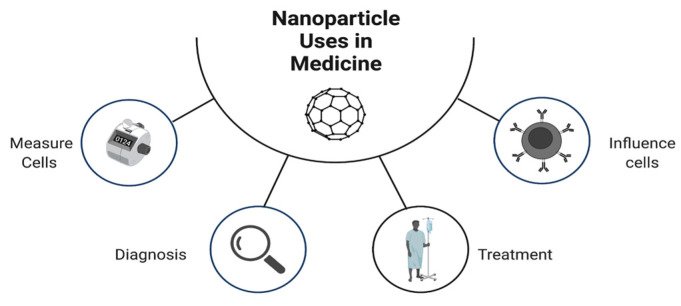
Main uses of nanotechnology in medicine.

Nanotechnology, which is the near-atomic manipulation of matter to create new structures, materials, and technologies, has found a home in medicine (Figure 4). Nanoparticles are unique in that due to their size (from 1 to 100 nanometers) and shape they exhibit increased capabilities that are not afforded to other tools used in medicine [36]. Similar in size to antibodies, membrane receptors, nucleic acids, and proteins, nanoparticles provide a variety of uses due to their electrochemical, optical, magnetic, catalytic, and thermodynamic properties [37,38]. Furthermore, nanoparticles can be outfitted with a “bioorganic interface” such as biopolymers or collagen that facilitate biocompatibility. Advancements in nanotechnology are used in imaging agents, drug delivery carriers, and radiosensitizers in radiation, proto, or photodynamic therapies [39,40,41]. While this is not a complete review of the potential uses of nanotechnology in medicine, several other comprehensive reviews have been published [42,43]. For the purposes of how nanotechnology can be used in the context of osteomyelitis, the focus of this review will be on diagnosis and treatment.

## 11. Nanotechnology in Diagnosis

**Figure 5:**
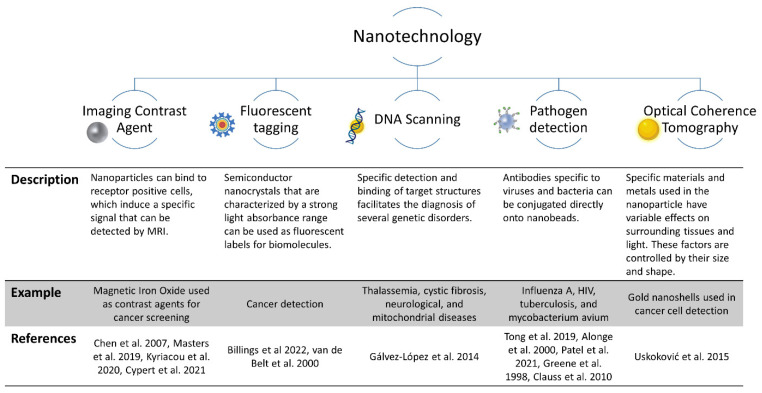
Summary of different types nanotechnology that can be used for disease diagnosis [45,47,48,49,50,51,52,53,54,55,56,57,58].

Although classically used to diagnose cancer, the use of nanoparticles in the field of clinical diagnostics (nanodiagnostics [44]) has been expanding to detect a wide range of diseases and provide clinically relevant information useful in treatment and diagnosis, particularly for those conditions that are difficult to detect early in their pathogenesis. As diagnostic technologies advance, the need for increasingly safe and sensitive methods has allowed nanotechnology to take its place among other traditional diagnostic tools and even improve them (Figure 5) [45,46]. This is particularly important for imaging.

*Nanotechnology in imaging-based diagnostics:* When compared with conventional probes for imaging contrast, nanotechnology provides several unique benefits including the capability to manipulate the physical (i.e., size and shape) as well as the biological (i.e., modifying the surface of targeting or to increase circulation time) properties. In fact, they can be dressed to provide multimodal imaging and therapeutic advantages [59].

*X-ray diagnostics:* Gold nanoparticles (AUNp) have been extensively studied as contrast agents for a variety of biomedical imaging techniques [60]. Au nanoparticles are particularly suited to the task as they have been shown to be biocompatible with easily functionalized surfaces, documented colloidal stability, and excellent X-ray attenuation. Zhang et al. [61] investigated the use of AUNps as a damage-specific X-ray contrast agent. Targeted Au nanoparticles were shown to bind damaged bovine cortical bone tissue. The investigative team concluded that Au nanoparticles were a “promising candidate” as an X-ray contrast agent targeting damaged bone tissue. Furthermore, it should be noted that Au nanoparticle-based contrast agents provide higher contrast than iodine-based contrast agents as well as having longer circulation times and decreased risks of negative side effects [62,63]. Although not currently used in the diagnosis of osteomyelitis, Au nanoparticle IV contrast may provide a method of earlier X-ray-based identification of bone damage, allowing further workup and preventative treatment.

*MRI diagnostics:* MRI is an extremely useful tool for identifying osteomyelitis and is quickly becoming the gold standard as it is able to delineate the extent of cortical destruction, evaluate bone marrow abnormalities, assess soft tissue inflammation, and determine ischemia [62,63]. The use of an IV contrast agent amplifies the diagnostic value of MRI. While not improving the initial detection capability of osteomyelitis on MRI, the inclusion of a contract agent enhances the power of this imaging modality as it improves the distinction between phlegmon, necrotic tissue, and abscesses, which may help to further characterize and stage a bony infection. The use of superparamagnetic iron oxide-based nanomaterials (SPION) as contrast agent provides an opportunity to bypass the issues associated with traditional gadolinium-based IV contrast agents [64]. In analyzing the safety of SPION, it was found that they are primarily cleared from the bloodstream by macrophages in the reticuloendothelial system (RES) and slowly degraded in lysosomes [65].

While this field of technology is ever expanding as both new and old techniques as well as nanostructures are refined, the mechanism of most of these technologies is based on the specific interaction of the particle with a molecular biomarker of disease. As nanotechnology continues to evolve, advancing the medical profession’s capacity to diagnose disease, many more technologies and uses will arise.

## 12. Use of Nanotechnology in Diagnosis of Osteomyelitis

Diagnosis of *S. aureus*, which is the primary causal bacteria in osteomyelitis infections [3], is prone to delays due to a lengthy amount of time necessary to run the test and low sensitivity [66,67]. Additionally, in the case of chronic infections, cultures may be negative based on the genetic and phenotypic alterations (e.g., biofilm growth with senescence and host cell infiltration). Thus, a need exists for improved diagnostics and several different nanotechnologies may fit the bill.

*Quantum dots* (QDs): QDs are a promising alternative to traditional methods and an emerging nanotechnology tool for the sensitive diagnosis of bacterial infections [68]. Specifically, QDs have been shown to be capable of detecting *S. aureus* through a quick and efficient process in which the QD acts as a biosensor that can be modified with *S. aureus*-specific antibodies to improve the specificity.

Cui et al. found through the development of a new optical fiber probe-based QD that their methods met the needs for rapid and accurate *S. aureus* detection [69]. Their method showed detection between 1 × 10^3^ CFU/mL and 1 × 10^4^ CFU/mL in as little as 2 h. This compared with bioluminescence imaging (another non-invasive method for the detection of *S. aureus*-induced osteomyelitis) which was able to detect a minimum of 7.8 × 10^5^ CFU/mL [70]. Development of these innovative uses of QDs to identify *S. aureus* may also pave the way for the sensitive detection of osteomyelitis secondary to *S. aureus*. Furthermore, this technology could be expanded beyond the detection of *S. aureus* to other pathogenic organisms by altering the QD probe’s surface-bound antibodies. In fact, this ability may be critical for the detection of complex polymicrobial infections that are becoming increasingly common.

Other pathogens implicated in osteomyelitis have also been identified with nanotechnology including but not limited to *Streptococcus* spp., *Enterobacter* spp., and *Enterococcus* spp. [71,72,73,74]. Carbon dots, a close cousin of the QD, have been used to identify *P. Aeruginosa* [75]. Despite these promising results, nanotechnology is not the diagnostic standard of practice for identifying bacterial infection in osteomyelitis. Nevertheless, there are definite diagnostic advantages to its application; QD and QD-like technologies may be extremely useful tools to not only diagnose osteomyelitis in the future, but also for the surveillance of pathogens after treatment, a critical need in the face of rising antibiotic resistance [73].

*SPION:* The use of IV contrast, while not improving the detection of osteomyelitis using MRI, improves the distinction between phlegmon, necrotic tissue, and abscesses which may help to further characterize the bony infection. A current challenge in using Gadolinium-enhanced contrast while visualizing areas of inflammation within bone parenchyma is in differentiating between specific causal pathologies [76]. Gadolinium-enhanced contrast is unable to differentiate osteomyelitis from sterile inflammation and from bone metastasis [77,78]. The unique application of nanoparticles to diagnose osteomyelitis was demonstrated by Fukuda et al. [79]. They observed that the use of SPION as a contrast agent can distinguish osteomyelitis from bone metastasis when the radiographic signals are analyzed. A different study investigating the use of ultra-small superparamagnetic iron oxide nanoparticles (USPION) showed their capability to specifically measure and identify macrophage activity to aid in the identification of areas of vertebral osteomyelitis [80]. Thus, in a diagnostic setting, SPION IV contrast use in MRI and CT may provide improved capability to diagnose and treat osteomyelitis quickly with fewer complications.

While nanotechnology provides a wide range of extremely useful tools and possibilities that can aid in the diagnosis of osteomyelitis, the field is continuously seeing new innovations and the repurposing of current technologies and agents. Further research and study of specific nanoparticle applications in the diagnosis of osteomyelitis is not only warranted but crucial as it may improve patient experiences as well as the long-term health and safety outcomes of treatment.

## 13. Nanotechnology in Treatment

Novel treatment methods for osteomyelitis may hold the key to improving osteomyelitis patient outcomes and a reduction of recurrent infection rates. One novel treatment that has shown efficacy with diagnosis and treatment in similar diseases is nanotechnology. Nanotechnology application in osteomyelitis diagnosis and treatment has yet to be fully explored and is an area that could benefit from more extensive focus as outlined below.

In a similar way that nanoparticles contribute to diagnosis by specific interactions with targeted tissues, they also facilitate extremely accurate drug delivery. Nanotherapies can be administered either parenterally or orally [81], since nanoparticles are capable, by means of their design and administration, of traveling directly to the site of action to elicit the desired, targeted effect. Due to this increased precision, the side effects of nanotherapy are lowered significantly. This approach to treatment not only decreases pain and other side effects for the patient but may ultimately be more cost effective [82]. Treatment through nanotechnology extends beyond the capability to deliver drugs, it also includes infrared thermal ablative methods [83], photodynamic therapy [84,85,86], and photothermal therapy [87]. The diversity of nanoparticle applications is progressing rapidly. Areas of medicine that may benefit from nanotherapy-based drug delivery include cancer, diabetes, infectious diseases, neurodegenerative diseases, blood disorders and orthopedic problems [88].

## 14. Nanotechnology in Osteomyelitis Treatment

**Figure 6:**
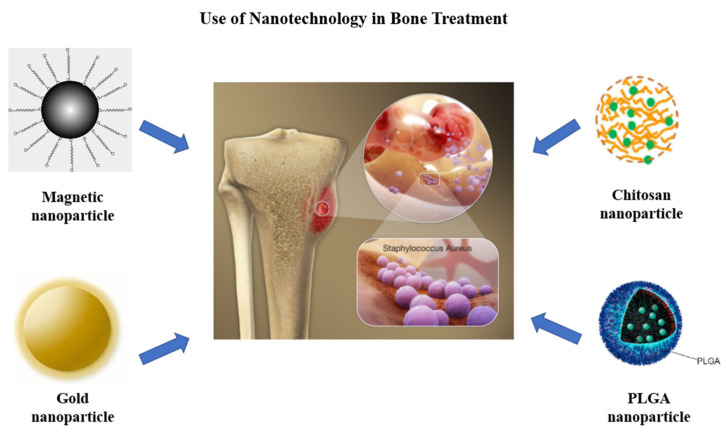
Uses of various types of nanoparticles in the treatment of osteomyelitis. Created with BioRender.com.

Effective treatment with nanotechnology likely will not occur in a vacuum and will require bulk material, both synthetic and natural, advancements as well (Figure 6).

*Use of Synthetic Biocompatible Materials*: Synthetic materials are advantageous due to the ability to control the chemical and physical properties of the material in order to, hopefully, direct the patient’s biological response. These materials include an impressive array of properties, ranging from degradable polymers [89] for tissue regeneration to the non-degradable metals, which provide support to the injured tissue/bone. As these materials are cost effective, their usage and variety is continuing to expand in the treatment of bone infections. A thorough review of this vast array of orthopedically suitable synthetic polymers is beyond the scope of this paper; however, a few key polymers and their applications are highlighted below [90,91,92].

*Poly(lactic-co-glycolic) acid (PLGA):* PLGA has garnered considerable attention for biomedical applications due to its tailorable bio-degradation rate as well as its status as GRAS (generally regarded as safe) and approved by the United States Food and Drug Administration [93]. Posadowska et al. prepared gentamicin-loaded PLGA nanoparticles for ultimate use as a novel therapy to treat osteomyelitis [94]. These PLGA nanoparticles were found to have in vitro drug release for 35 days and showed promising antibacterial effects against *Staphylococcus aureus* and *Staphylococcus epidermidis* in agar diffusion tests. Similarly, Pillai et al. prepared nafcillin-loaded PLGA nanoparticles to deliver the antibiotic to *Staphylococcus aureus*-infected mouse osteoblasts. The data showed significant reduction in intracellular *S. aureus*, suggesting a particularly effective therapy for chronic osteomyelitis [95].

*Polyanhydride:* Polyanhydride is one of several biodegradable synthetic polymers showing therapeutic effectiveness for bone infections [96]. In 2002, Abbott laboratories published the research data pertaining to their proprietary product containing polyanhydrides and gentamicin (Septacin) for the treatment of osteomyelitis. Human in vivo trials have shown that polyanhydrides in Septacin could retain high therapeutic concentration at the implantation region and low systemic concentration for gentamicin [97]. The blend of polyanhydrides and polylactide were compressed into beads along with ofloxacin. This combinatorial polymer formulation inhibited the growth of *S. aureus*, *E. coli*, and *P. aeruginosa* within 89 days of the treatment. In vivo studies conducted on rabbits showed that the blend of polymers maintained the optimum concentration of antibiotic at the bone [47].

*Bone cements:* Use of bone cement as a drug carrier is perhaps the most well-known and longest approved delivery vehicle for the local delivery of antibiotic to the bone. Poly(methyl methacrylate) (PMMA) beads, calcium sulfate and calcium phosphate are the most common bone cement materials used for antibiotic delivery. While an extensive discussion of these materials is beyond the scope of this review, several good reviews have been published recently, including reviews by Masters et al. in 2019 [48], Kyriacou et al. in 2020 [49], Cyphert et al. in 2021 [50], and Billings and Anderson in 2022 [51]. Within these materials, several antibiotics including gentamicin [52,53], ceftriaxone [54], tobramycin [55], and vancomycin [56] have been successfully loaded and clinically tested. A significant disadvantage of many bone scaffold materials as well as other bone graft void filling materials is that they can serve as a nidus for infection, offering a surface for the attachment of bacteria and fostering the development of a biofilm [57]. Although these materials may serve as a homing beacon for bacterial seeding, they can also often promote host bone ingrowth. It is the delicate balance of these functions that must be designed to favor host cell seeding in the “race for the surface”.

The use of nanomaterials as a bone scaffold may tip this balance in favor of the host. In contrast to completely synthetic cements, nano calcium phosphate (CAP) appears to be a more natural choice as it is a natural component of the mineral composition of bone, and thus, one of the safest and most biocompatible nanomaterials with the least toxicity [58]. The microstructure of nano hydroxyapatite (n-HA) is similar to that of natural bone tissue and has been modified in various ways in the literature including polyurethane, PCL for effective delivery of antibiotic and bone healing. Even without modification, it has been demonstrated that n-HA can be synthesized under conditions to resist *S. epidermidis* seeding and biofilm formation [98]. Nano structured silicon-substituted HAp powder was deposited as a coating on titanium implants and studied for osteoblastic cells activity. The prepared coatings were dense, crystalline and found to improve osteointegration [99]. Levofloxacin-encapsulated mesoporous silica combined with an n-HA/polyurethane bioactive composite scaffold was synthesized and investigated in the treatment of chronic osteomyelitis induced in rabbit tibia [100]. The bioactivity of the n-HA/PU combined matrix was increased and the incorporation of 40 wt% n-HA particles in polyurethane (PU) observably promoted new bone formation and bone repair while also controlling inflammation. It was observed that the biocomposite started to degrade 12 weeks after implantation.

*Scaffolds:* While scaffolds themselves can be bioactive and resist bacterial colonization, drug delivery can also be targeted either physically, chemically or biologically. Local delivery can be achieved either by antibiotic-treated scaffolds or an injectable formulation with the latter being more efficient for penetration into the bone tissue while simultaneously being less invasive. In this regard, the utilization of nanomaterials may offer improved therapeutic effects by the incorporation of osteoinductive and osteoconductive materials aiding in the proliferation and differentiation of bones, potentially restoring joint function [101]. Nanocomposite compounds provide a novel and flexible platform for incorporating drugs and other bioactive components into scaffolds with their large surface area, higher reactivity and extraordinary chemical, physical and biological properties. Nanoparticles can also minimize the dosage and be modified for targeted therapy.

*Bioactive glass*: In the last decade, the usage of bioactive glass, a type of bioceramic, has been extensively investigated for tissue engineering and other pharmaceutical fields. Bioactive glass contains calcium, phosphate and silicon ions, which are released in physiological conditions to aid the osteoblast proliferation and differentiation. In fact, several studies have used bioactive glass as a composite for bone regeneration. Recently, a bilayer membrane composed of poly (lactic-co-glycolic acid) (PLGA) and micro-nano bioactive glass was reported to show tremendous potential for guided bone regeneration [102]. Zhang et al. created a 3D printed bioactive glass with integrated chitosan nanoparticles to release either Nel-like Type I molecular-1 DNA (pDNA-NELL1) and/or bone marrow mesenchymal stem cells (BMSCs) to fill an oral defect. Using this composition, they found enhanced bone regrowth, noting that the new bone had similar properties (i.e., mass, density, hardness, and structure) as the host bone [103].

Several other compositions with micro-nano bioactive glass are currently being developed [104,105,106]; however, they are not discussed here due to a lack of in vivo evidence of their osteogenic abilities. Each of these bioglass composites [107], particularly composites with a nanoscale component, seem to strongly support bone regeneration; however, based on the work of Drago et al., which demonstrated that bioactive glass granules have an innate antibacterial function against osteomyelitis causative microorganisms even without the addition of any antibiotics, these materials may find particular utility in the treatment of destructive bone osteomyelitis [108]. Others have used bioactive glass to deliver antibiotic locally to the bone. Jia et al., evaluated teicoplanin (TEC)-loaded borate bioactive glass and tested its ability to release TEC in vitro and to cure methicillin-resistant *Staphylococcus aureus* (MRSA)-induced osteomyelitis in a rabbit model [109]. The bioglass implant showed a slower decrease in strength compared to the calcium sulfate implant; moreover, teicoplanin was found to be more effective when released from bioglass than when administered intravenously. Hasan et al., has proven that vancomycin-loaded bioactive glass putty is effective in treating a rat model of osteomyelitis. This novel bioactive glass bone-void-filling putty has shown in vitro antibacterial activity for 6 weeks and also supported bone growth in an osteomyelitis-induced rat model [110].

*Use of Natural Biopolymers and Composites:* Gelatin is not the only natural polymer that has been explored as a scaffold or additive to enhance bone regeneration and there is currently a trend away from synthetic polymers toward green biopolymers [111]. Collagen, hyaluronic acid (HA), carboxymethyl cellulose (CMC), and chitosan are some of the most studied natural polymers for bone regeneration, a topic thoroughly reviewed recently by Filippi et al. [112]. Collagen has specifically been fabricated into a drug-eluting sponge for the treatment of acute and chronic osteomyelitis, with recent findings indicating that not only was the gentamycin-infused collagen scaffold biodegradable and bioresorbable, but it also reduced bacterial counts more significantly than its PMMA counterpart [113]. Alternatively, collagen has also shown promise as a nanocomposite. A heparinized biocomposite of nanohydroxyapatite/collagen granules was capable of mimicking the composition of bone and promoting bone regeneration while simultaneously releasing vancomycin for 19 days in an osteomyelitis model, making this composite a versatile material for the treatment of osteomyelitis [114].

Collagen is not the only natural, structural protein to find utility in the treatment of osteomyelitis. Chitosan, the second most abundant biopolymer in nature, has also been explored as a bone-regenerating scaffold and/or drug-delivery vehicle for the treatment of osteomyelitis. In one particularly unique use of this biopolymer, Aimin et al. prepared gentamicin-loaded chitosan bars for the treatment of osteomyelitis. The innovative chitosan bar was implanted in the proximal end of a rabbit’s tibia and was able to maintain a therapeutic concentration of gentamicin for 8 weeks without any side effects [115]. Pujiang shi et al. prepared gentamicin-loaded composite microspheres formulated with chitosan, nanohydroxyapatite and ethyl cellulose. These microspheres were capable of releasing their drug payload for 45 days in a rabbit osteomyelitis model with no recognized cytotoxic effect [116]. While these were microspheres, others have found similar success with chitosan nanoparticles. Tao et al., engineered chitosan complex nanoparticles with an assembly of positively charged ammonium chitosan and negatively charged carboxylated chitosan [117]. These electrostatic adsorption-driven nanoparticles formed a thermosensitive hydrogel with high vancomycin encapsulation efficiency for the treatment of osteomyelitis. The chitosan nanoparticle-based hydrogel was shown to release its vancomycin payload at a sustained rate for 28 days. Additionally, this novel chitosan formulation has promoted the proliferation of osteoblasts.

While chitosan and collagen may be abundant, silk fibroin (SF), such as that produced by the mulberry silkworm, *Bombyx mori*, and by spiders *Nephila clavipes* and *Araneus diadematus*, is more mechanically robust, which may make it more suitable for orthopedic applications. Additionally, SF has been explored as a biomaterial due to its biocompatibility, low immunogenic response, low bacterial adhesion, mechanical functionality, and tunable degradation. Its β-sheet structure gives it an easily tunable architecture that allows processing into various structures, such as hydrogels, fibers, membranes and microspheres [118,119,120]. Silk fibroin incorporated into HA has been shown to be superior to plain HA because of its porous structures that provide better transportation of blood and body fluids for metabolism and growth of bone as HA nanoparticles are uniformly distributed on SF nanofibers and considered to be helpful in proliferation and cell adhesion [121]. Alternatively, vancomycin-loaded silk fibroin nanoparticles (VSFNP) loaded on SF scaffolds were investigated in a rat osteomyelitis model. Although bone abscesses were reduced in the VSFNP group, nanoparticles loaded on SF scaffolds reduced infection effectively compared to the VSFNP particles alone. However, bone regeneration was not observed [122]. Similar to silk fibroin, spidroins, or spider silk proteins, also have gained importance as biomaterials in recent years. Although most of their properties are similar to silk fibroin, spidroins have a block copolymer structure, which can be easily customized according to rates of degradation. Furthermore, in vivo investigation has demonstrated that, upon degradation, non-toxic byproducts are formed. Capitalizing on this tunable nature, Mulinti et al. developed vancomycin-loaded silk nanospheres that could be triggered to release their drug payload. By modifying a recombinant spider silk protein with a thrombin sensitive peptide, a nanosphere was formed to encapsulate vancomycin for the treatment of septic arthritis secondary to osteomyelitis. When tested in both in vitro and in vivo using a rat disease model, the nanospheres were able to effectively clear the bacteria from the infection site; no bone resorption or bone regeneration was observed based on the limitations of the rat model chosen [123].

*Incorporation of Metallic Nanoparticles*: Metal-coated implants and drug-eluting implants offer a promising approach for localized antimicrobial activity and sustained antibiotic drug delivery. Titanium, cobalt and stainless steel are the most commonly used implant materials. However, the bulk implant material alone is most often non-biodegradable with limited antimicrobial and bone-regenerating activity. Therefore, implants are frequently doped with other heavy metals for a better outcome of treatment.

Silver nanoparticles are the most commonly studied material due to their intrinsic antimicrobial property, leading them to be used to treat various wound infections [124,125,126]. Although there are many studies reporting the antimicrobial effect of silver ions and a lower rate of antimicrobial resistance, high concentrations of silver may lead to severe cytotoxic effects [127,128,129]. Therefore, addition of a secondary ion or chemical has been shown to reduce the toxicity of silver while also maintaining the antimicrobial activity. The addition of zinc (Zn) to silver nanoparticles was found to be more effective as Zn can promote osteogenic functions by enhancing cell proliferation, differentiation, and osteoblast marker gene expression due to its structural similarity to a large number of proteins [130]. Silver nanoparticles are commonly combined with hydroxyapatite to develop scaffold materials which have shown excellent biocompatibility and osteoconductivity [124,131].

Lu et al. developed nanosized titanium (TiO_2_) and silver-co-substituted nano-hydroxyapatite/polyamide-66 composite scaffold materials (or TA-nHP66) and tested them in a rabbit model of experimental osteomyelitis. The nano scaffold was shown to exhibit potent antibacterial activities against both *E. coli* and *S. aureus* bacterial cells while also supporting pre-osteoblastic cell proliferation. This nano scaffold was also shown to have minimal systemic toxicities with excellent biocompatibility. Magnesium can also be co-substituted with silver and hydroxyapatite to enhance bone resorption ability and the new bone tissue generation process [132]. The addition of Mg was shown to reduce the toxicity of silver thereby increasing the cell viability. Geng et al. evaluated the antibacterial and biocompatibility properties of strontium (Sr) co-substituted in silver-hydroxyapatite coated bone implants [129]. Sr was shown to not only enhance pre-osteoblastic cell proliferation; it also increased bone formation, the number of bone-forming sites and bone mineral density and inhibited osteoclast activity [133]. The addition of other inorganic materials such as copper (Cu) and boron (B) to silver nanoparticles has also been explored, which shows the longevity of antimicrobial properties [134]. Thus, various metallic nanoparticles have been successfully developed for the effective treatment of osteomyelitis.

*Incorporation of Magnetic Nanoparticles:* While metallic nanoparticles often have innate antimicrobial activity due to leachable ions, magnetic nanoparticles also offer advantages in the treatment of osteomyelitis. The use of magnetic nanoparticles for targeted drug delivery has been well established in oncologic applications for the accumulation and retention of chemotherapeutic drugs in a tumor. Magnetic nanoparticles have also been explored for the treatment of osteomyelitis in order to target the drug to bone tissue and also to prevent elimination from the reticuloendothelial system [135]. Superparamagnetic iron oxide nanoparticles were used together with vancomycin for the treatment of osteomyelitis in a rat disease model. The authors found that the use of magnetic nanoparticles increased the efficiency of the treatment because of hyperthermia generated by the particles, which eradicated the biofilm [136]. Hyperthermia may not be the only advantage of magnetic nanoparticles. In another study, iron oxide nanoparticles coated with hydroxyapatite were designed and studied for their biocompatibility and biodegradability [137]. Gentamycin-loaded magnetic gelatin nanoparticles were formulated for the local treatment of osteomyelitis [138], showing that the rats treated with the drug-loaded magnetic nanoparticles recovered faster in comparison with the free drug. Since gelatin is a natural polymer, it has an added advantage of biocompatibility and biodegradability.

## 15. Nanotoxicity and Nanoparticle Safety in Treatment

Although nanoparticles offer various advantages over their parent material, their small size poses various toxicities that are not evident with larger particles [139]. Some of the important particle parameters that may influence their toxicity include size, surface chemistry, surface coating, bulk chemical composition, etc. Nanotoxicity has limited the clinical translation of nanoparticles in a variety of applications. Silver nanoparticles have been shown to be cytotoxic to osteoblasts and osteoclasts, damaging DNA and resulting in a deleterious effect on the biocompatibility of orthopedic implants [140,141]. Specifically, particles were found to be cytotoxic to osteoblasts in a dose-dependent manner, potentially due to the noted increase in oxidative stress [142]. Beyond the musculoskeletal system, the ability for some nanoparticles to bypass the normal host phagocytic defense and gain access to the blood and brain has led to significant concerns about the safety of nanoparticles. Additionally, magnetic nanoparticles typically containing iron oxide have raised concerns about cytotoxicity and other acute adverse events, such as nephrogenic systemic fibrosis, the formation of apoptotic bodies, and inflammation [143]. Other particles composed of polymers such as PLA and PLGA can yield cytotoxic acidic degradation products [144]. The interaction of nanoparticles with various fluids, cells and tissues may trigger significant biological responses and a thorough investigation needs to be conducted both in vitro and in vivo in order to study nanotoxicity and establish a safety profile for these nanoparticles.

## 16. Therapies for Osteomyelitis in Clinical Trials

Nanotechnology is an active and expanding area of research with a multitude of clinical trials underway. New research is expanding into some uses that consist of filling bone voids after resections and facial reconstruction [145,146], re-infection prophylaxis [147], and the treatment of chronic health issues such as osteoarthritis [148]. This movement of research and product development aimed toward treating a multitude of illnesses coincides with the ability for size to be determined utilizing state of the art manufacturing techniques to tailor particle size down to the angstrom scale. Among this multitude of diseases, treating osteomyelitis with nanotechnology is of particular interest.

Even though nanotechnology research is on the rise, there seems to be a limited body of clinical trials occurring that pertain to the treatment of osteomyelitis. With no current clinical trials listed on the National Institute of Health (NIH) Clinical Trials website underway for nanotechnology to treat osteomyelitis, it is difficult to evaluate progress within the field, chronic or acute. The NIH clinical trials website lists 69 current trials with nanotechnology. However, when narrowing the search down to “recruiting,” “not yet recruiting”, “active not yet recruiting”, and “enrolling with invitation”, only 19 studies remain on the list, illustrating that while there is progress in the field, there is not an abundance of clinical research ongoing.

While there is definitely a need for expansion, there are a few studies of note that seem promising when considering the use of nanotechnology to treat osteomyelitis. After searching clinical trials using sources such as the National Institute of Health, the World Health Organization, CenterWatch, and the Mayo Clinic, there were only three main studies that even remotely fit the criteria: “Post-operative Pain Reduction After Application of Three Intracanal Medicament Within Necrotic Root Canals and Pulp” [149], “Rate of Bony Fusion Using NanoBone Synthetic Bone Graft Versus Local Autologous Bone Graft (BONE)” [145], and “Evaluation of the Efficiency of the Bone Substitute Cerament-G Locally Delivering Gentamicin in the Treatment of Chronic Osteomyelitis of Long Bones (CONVICTION)” [150].

In the trial examining pain reduction [149], silver nanoparticles are used in conjunction with the standard of care, calcium hydroxide, in patients that have necrotic pulp after a root canal. This study is hoping to show that silver nanoparticles incorporated into the traditional calcium hydroxide paste will show superior control in the patient’s pain when compared to the calcium hydroxide paste alone. While this study examines utilization of nanotechnology to infuse bone and the surrounding soft tissue in the hopes of reducing pain, it does directly address the infection of the necrotic root itself, although, based on the antimicrobial molecular mechanism of silver, that may be a side benefit.

In the trial of synthetic bone graft being compared to an autologous bone graft for patients with degenerative disk disease or grade-3 spondylolisthesis [145], all of the participants received posterolateral spinal fusions, with an autologous graft on their left side and NanoBone being used on their right. Patients are being evaluated with spinal CT scans at one year postfusion surgery. This trial is still in progress and is set to be concluded toward the end of 2024, so the results will not be available any time soon. Again, this is another promising study for nanotechnology but the goal of treatment is not aimed at osteomyelitis. This product has a nanocrystalline structure that acts as an osteoconductive scaffolding structure and could potentially be appealing in the future as a conduit for postsurgery bone enhancement.

As opposed to the two previous trials that did not deal directly with osteomyelitis, but did involve an aspect of nanotechnology, the third trial is directly addressing the treatment of osteomyelitis; however, Cerament-G is not mentioned as nanotechnology specifically in either of the trials nor do the dimensions of the product, as stated on the product fact sheet [151], meet the sizing guidelines of 1–100 nm [152] to be a nanoparticle product. Nevertheless, Cerament-G uses gentamicin-impregnated synthetic bone material to treat osteomyelitis. The product has been used in trials for treating chronic infection in long bones [150] and in surgically revised long bone infections [147].

These trials, while demonstrating there is a need and desire for products that treat challenging osteomyelitis infections, show that there are not many new products in the clinical trials pipeline. The lack of trials for osteomyelitis, particularly for products that integrate nanotechnology, is disappointing considering the relatively strong preclinical data in the literature. However, a few considerations should be kept in mind when evaluating the clinical trial data for osteomyelitis. Clinical trials have long time frames between the start and completion of the trial; hence, many trials either do not have results yet, such as the NanoBone clinical trial mentioned, or the results were never updated, as in the trial utilizing Cerament-G in surgically revised long bones (completed in 2015). This is a problem that extends more broadly beyond osteomyelitis clinical trials since there is no requirement for NIH to be notified of why trials were ended and no follow up and public disclosure for studies terminated early. Without updates on trial progress, particularly in the case of early termination due to negative or adverse results, assessing the state of nanotechnology in osteomyelitis is difficult at best. This lack of transparency in the context of current treatment protocols that often require multiple antibiotics, also demonstrates that there is an unmet need for osteomyelitis treatments. This may also be a reflection of the complexities of the condition, all too often compounded by a delay in diagnosis and treatment [153] or that altering the traditional standard of care is difficult. In fact, there may be no single nanoparticle to cure an infection and as with many other infections, a combination delivered locally may prove the best option.

## 17. Conclusions

Osteomyelitis is a challenging condition to treat. The underlying opportunistic pathogens have developed a multitude of strategies to avoid the host immune response and their physiological niche is protected against antibiotic onslaught by compromised vasculature. These complexities often lead to delayed diagnosis and inadequate clinical treatments. Nevertheless, new strategies are emerging to provide new scaffolds for host bone regrowth and local delivery of antibiotics. Translating nanotechnology, which has been so successful in oncological and other applications, to osteomyelitis will be pivotal in driving this advancement forward. Evidence is mounting that the inclusion of nanotechnologies in these strategies is not only safe but is particularly promising to both promote host bone regeneration and kill infection. Clinical trials are needed to translate these preclinical nanotechnology-based ideas to the patient’s bedside, allowing significant progress in the prevention and treatment of osteomyelitis.

## Figures and Tables

**Table 1:** Diagnostic criteria for osteomyelitis proposed by Schmidt et al.

**Diagnostic Procedure**	**Scoring**	**Diagnosis**	**Score (Add the Score for Each Procedure)**	
Clinical History and Risk Factors	Can score up to 6 points/procedure			
Clinical examination and laboratory test results (e.g., leukocyte counts, inflammatory markers, ESR, and CRP)				
		Class A—Safe	≥18 points	
		Class B—Probable	8–17 points	
Diagnostic imaging (e.g., ultrasounds, radiology, CT, MRI, nuclear medicine, etc.)				
		Class C—Possible but unlikely	≤8 points	
Microbiology analysis				
Histopathology				
A reliable diagnosis can only be made if at least 3 procedures are scored with 6 points.				

**Table 2:** Marais staging scale to integrate clinical, laboratory, and radiological findings.

	Grade	Characteristics
**Clinical**	Acute	
	Grade 1	Acute fulminating
	Grade 2	Sub-acute
	Grade 3a	Acute with insidious onset
	Grade 3b	Acute exacerbation of chronic
	Chronic	
	Grade 4	Chronic overwhelming
	Grade 5	Chronic diffuse with inflammation
	Grade 6	Chronic low grade extensive without inflammation
	Grade 7	Chronic localized lesion
	Grade 8	Non-infective pathology
**Laboratory Results**	Chronic	
	Grade 4	Increased WBC, neutrophilia, left shift and toxic granulation, decreased transferrin, procalcitonin > 2, increased platelets, abnormal RBC corpuscles
	Grade 5	Decreased Hb MCV and MCH, rouleaux formation
	Grade 6	Increased ferritin, decreased iron, decreased iron saturation, increased ESR
	Grade 7	Ferritin iron ratio > 7
	Grade 8	Normal
**Radiological Findings**		▪ Definite infection ▪ Probably infection ▪ Equivocal ▪ Probable cure or absence of infection ▪ Definite cure or absence of infection ▪ New bone lysis or sequestrum ▪ New periosteal reaction ▪ No change ▪ Sclerosis only ▪ Normal bone architecture

**Table 3:** Antibiotic regimens for the treatment of osteomyelitis.

Antibiotic:	Dosage:	Notes:
Nafcillin	9–12 g/day (6 individual doses) (IV administration)	Empiric antibiotic of choice Causative bacteria: *Staphylococcus aureus*
Penicillin G	4 million units every 6 h (IV administration)	Causative bacteria: *Streptococcus pneumoniae*
Vancomycin	30 mg/kg/day (2–3 doses) (IV administration)	Used for patients with penicillin-allergic reactions due to methicillin resistant *S. aureus* (MRSA) Causative bacteria: MRSA
Ceftazidime	2g every 8 h (IV administration)	Administered with an aminoglycoside IV for the first two weeks Causative bacteria: *Pseudomonas aeruginosa*
Ciprofloxacin	750 mg every 12 h (Oral administration)	Causative bacteria: Enteric gram-negative rods
Augmentin	875 mg every 12 h (Oral administration)	Causative bacteria: Mixed aerobic organisms
Clindamycin	600 mg every 6 h (Oral administration)	Causative bacteria: Anaerobes

## Data Availability

Not applicable.
